# Comparison of Substituting Ability of Nitronate versus Enolate for Direct Substitution of a Nitro Group

**DOI:** 10.3390/molecules25092048

**Published:** 2020-04-28

**Authors:** Yusuke Mukaijo, Soichi Yokoyama, Nagatoshi Nishiwaki

**Affiliations:** 1 School of Environmental Science and Engineering, Kochi University of Technology, Tosayamada, Kami, Kochi 782-8502, Japan; 235030n@gs.kochi-tech.ac.jp (Y.M.); yokoyama.soichi@sanken.osaka-u.ac.jp (S.Y.); 2 Research Center for Molecular Design, Kochi University of Technology, Tosayamada, Kami, Kochi 782-8502, Japan; 3 The Institute of Scientific and Industrial Research, Osaka University, Mihogaoka, Ibaraki, Osaka 567-0047, Japan

**Keywords:** conjugate addition, dihydrofuran, 1,3-dicarbonyl compound, enolate, isoxazoline *N*-oxide, nitro group, nitroketone, nitronate, nucleophilic substitution

## Abstract

α-Nitrocinnamate underwent the conjugate addition of an active methylene compound such as nitroacetate, 1,3-dicarbonyl compound, or α-nitroketone, and the following ring closure afforded functionalized heterocyclic frameworks. The reaction of cinnamate with nitroacetate occurs via nucleophilic substitution of a nitro group by the *O*-attack of the nitronate, which results in isoxazoline *N*-oxide. This protocol was applicable to 1,3-dicarbonyl compounds to afford dihydrofuran derivatives, including those derived from direct substitution of a nitro group caused by *O*-attack of enolate. It was found the reactivity was lowered by an electron-withdrawing group on the carbonyl moiety. When α-nitroketone was employed as a substrate, three kinds of products were possibly formed; of these, only isoxazoline *N*-oxide was identified. This result indicates that the substituting ability of nitronate is higher than that of enolate for the direct S_N_2 substitution of a nitro group.

## 1. Introduction

The nitro group is one of the important functional groups because of its unique chemical properties, which are useful in many compounds. The strong electron-withdrawing property of the nitro group reduces the electron density of the adjacent atom or double bond through both inductive and resonance effects. The increased electrophilicity facilitates nucleophilic addition to a nitroalkene, while the resulting anionic intermediate is stabilized by the nitro group. The nitro group also serves as a good leaving group. Nitroalkane undergoes E2 elimination of nitrous acid to afford C–C double bonds under basic [1,2,3,4] or acidic conditions [5,6]. Direct S_N_2 substitution is also sometimes observed [7], in which a nitrite ion is eliminated. The high acidity of an α-proton of the nitro group easily generates a nitronate, which possesses both nucleophilic [8,9] and electrophilic sites to serve as a 1,3-dipole [10,11]. Moreover, the nitro group is a precursor of amino and carbonyl groups by reduction and Nef-type reactions [12], respectively. The many properties of the nitro group have facilitated diverse applications. Recently, the complex/mixed properties of the nitro group have attracted considerable attention for the synthesis of multi-functionalized/polyfunctionalized compounds. In our previous work, α-nitrocinnamate served as a precursor of functionalized enynes via conjugate addition of an acetylide ion followed by elimination of a nitrous acid [1]. When α-chloro-α,β-unsaturated ketone is subjected to the reaction with cyano-*aci*-nitroacetate, intramolecular nucleophilic substitution of the chloro group by the nitronate ion occurs after Michael addition [13]. Based on these works, this study furthered this topic by studying the synthesis of functionalized heterocyclic compounds using a combination of conjugate addition of an active methylene compound and subsequent *O*-attack of the resulting nitronate/enolate which undergoes direct substitution of the nitro group. The substituting abilities of the nitronate/enolate were compared.

## 2. Results and Discussion

**Scheme 1:**
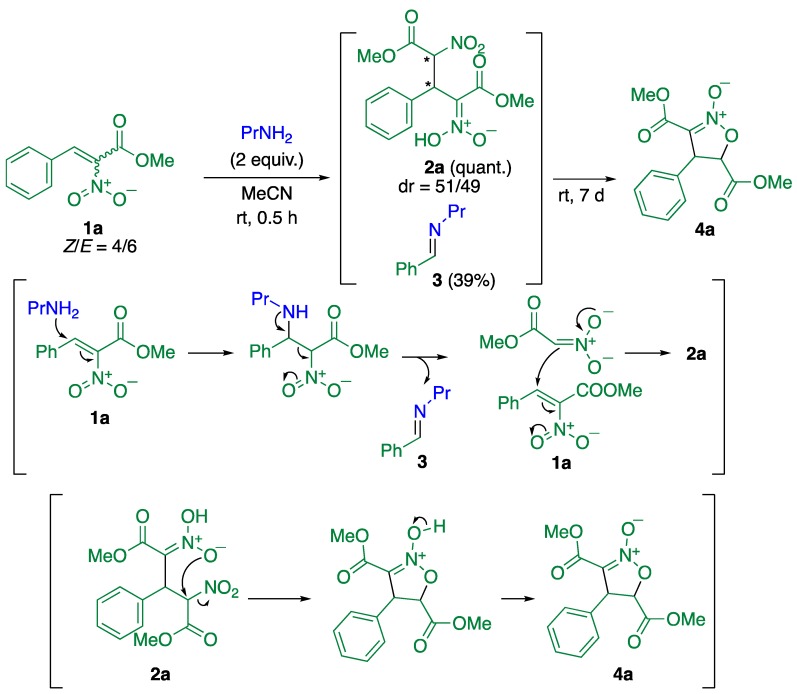
Reaction of α-nitrocinnamate **1a** with propylamine and the subsequent ring closure, with plausible mechanisms.

As a highly electron-deficient substrate, methyl α-nitrocinnamate (**1a**) was employed because of its easy availability via condensation of benzaldehyde and nitroacetate in the presence of piperidine hydrochloride with the removal of water as an azeotrope mixture. Indeed, the reaction of **1a** with propylamine quantitatively proceeded at room temperature in acetonitrile to afford product **2a** as a 1:1 mixture of diastereomers, which implies that two stereocenters were newly formed; however, the product was not an adduct of **1a** and propylamine. Based on spectral data of the reaction mixture, compound **2a** was confirmed to be an adduct of **1a** with methyl nitroacetate generated in situ. This reaction is thought to proceed as shown in Scheme 1. After conjugate addition of propylamine to **1a**, C–C bond cleavage forms nitroacetate [14]. Indeed, signals of 1-phenyl-*N*-propylmethanimine (**3**) were observed in the ^1^H NMR spectrum of the reaction mixture (see Supplementary Materials). The generated nitroacetate underwent the conjugate addition to another cinnamate **1a** to afford product **2a**. However, product **2a** could not be isolated by column chromatography because of its instability on silica gel. On the other hand, when the mixture of **2a** and **3** was left at room temperature without solvent for 7 days, ring closure proceeded to afford isoxazoline *N*-oxide **4a**, in which the nitronate underwent the direct substitution of the nitro group to form an isoxazoline framework (Scheme 1).

There have been several studies of the formation of isoxazoline *N*-oxides from α-nitro-α,β-unsaturated esters with C–H acids such as secondary nitroalkane [15], (ethoxycarbonylmethyl)dimethylsulfonium salt [16], (ethoxycarbonylmethyl)ammonium salt [17], (ethoxycarbonylmethyl)pyridinium salt [18], α-halomalonate [19], and α-iodo aldehyde [20]. β,β-Dimethoxynitroethene is also usable as a nucleophile in this protocol [21]. Among these, only two methods employ a nitro group as a leaving group [15,21]. In these reactions, the nucleophilicity of the nitronate ion is relatively high. To the contrary, nucleophilicity of the nitronate in **2a** is considered to be lower due to the electron-withdrawing ester functionality.

**Scheme 2:**
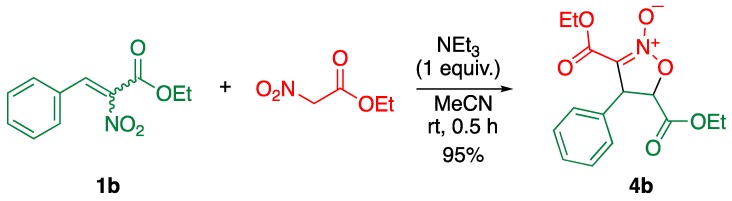
Synthesis of isoxazoline *N*-oxide **4b**.

Thus, ethyl α-nitrocinnamate (**1b**) was allowed to react with ethyl nitroacetate in the presence of triethylamine. It was confirmed that the successive conjugate-addition/ring-closure reactions efficiently proceeded in one pot to afford isoxazoline *N*-oxide **4b** [22] (Scheme 2). This result prompted the study of α-nitrocinnamate **1b** reactions with other active methylene compounds such as 1,3-dicarbonyl compounds, because the nucleophilicity of the enolate ion is considered to be lower than that of the nitronate ion.

Although two studies on the reactions of **1b** with β-keto esters were found in the literature [23,24], they were not conducted under same conditions (one reaction was conducted in the presence of tetrabutylammonium bromide). In order to compare the reactivity systematically and to generalize this protocol, the same reaction conditions should be used. Therefore, 1,3-dicarbonyl compounds **5a–f** were subjected to reactions with **1b** in the presence of triethylamine at 60 °C for 3 h in acetonitrile (Table 1). Cyclization efficiently proceeded to produce a furan derivative **6a** [23] with 92% yield when ethyl acetoacetate was employed as a substrate (entry 1). The nucleophilicity of the enolate of the ketone functionality was higher than that of the ester functionality because of the electron-withdrawing inductive effect of the ethoxy group. Next, the acetyl group of **5a** was replaced with a trifluoroacetyl group. In this case, decreasing the nucleophilicity of the enolate ion was more effective than increasing the acidity of the methylene group, which produced 5-(trifluoromethyl)-2,3-dihydrofuran **6b** with a lower yield (entry 2). When benzoylacetate **5c** was reacted under the same conditions, the cyclization occurred without significant effects from the steric hindrance of the phenyl group to furnish the corresponding dihydrofuran **6c** (entry 3). Diketone **5d**, acetylacetone, exhibited higher reactivity than keto esters **5a**–**c**, and yielded **6d** [24] quantitatively (entry 4). Cyclic diketone **5e**, 1,3-cyclohexanedione, also underwent the reaction efficiently to produce bicyclic furan **6e**, which was not influence by steric strain (entry 5) [23,25]. On the other hand, the formation of ester-substituted furan **6f** was not detected when diester **5f** was subjected to the reaction conditions because of the low nucleophilicity of the enolate ion (entry 6).

**Scheme 3:**
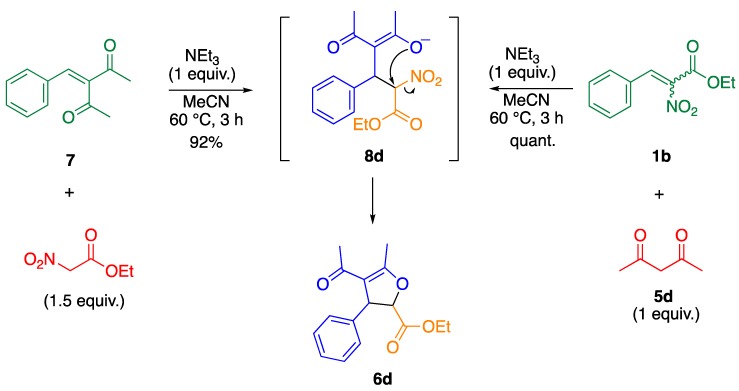
Synthesis of 2,3-dihydrofuran **6d** from either diacetylated styrene **7** or cinnamate **1b**.

In the reaction of cinnamate **1b** and diketone **5d**, the conjugate addition of enolate ion of **5d** to **1b** afforded adduct intermediate **8d**, from which 2,3-dihydrofuran **6d** was formed by the substitution of the nitro group. In other words, 2,3-dihydrofuran **6d** was also synthesized if intermediate **8d** was formed [24]. Indeed, when diacetylated styrene **7** was reacted with ethyl nitroacetate in the presence of triethylamine under the same conditions, a high yield of dihydrofuran **6d** was obtained (Scheme 3).

**Scheme 4:**
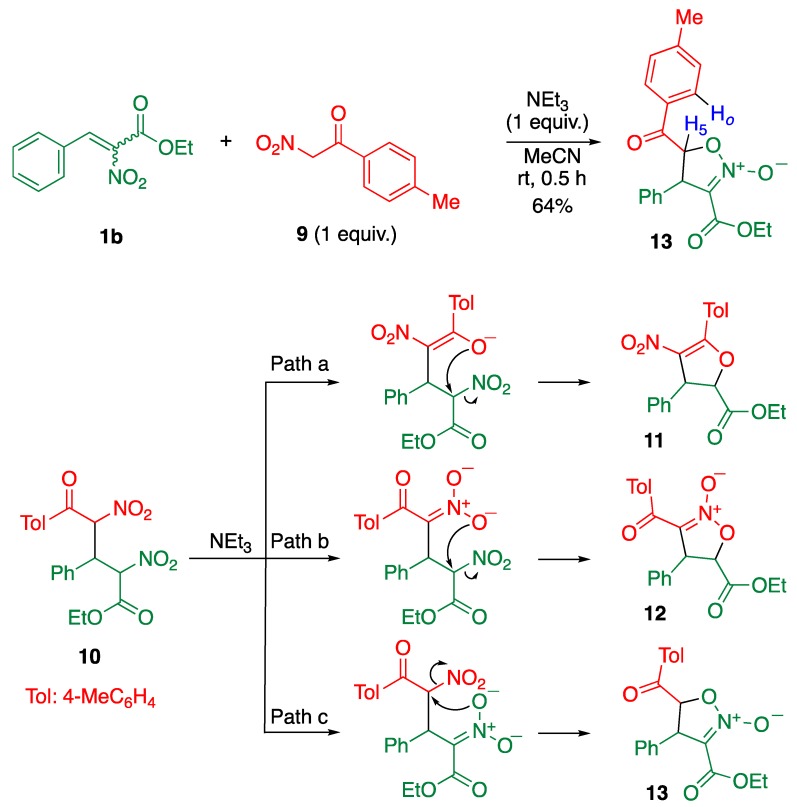
Reaction of cinnamate **1b** with nitroketone **9** and three possible products **11**–**13**.

Next, α-nitroketone **9** was employed as a nucleophile able to produce both an enolate and a nitronate ion, which facilitated the comparison of the substituting ability of these anions directly (Scheme 4). In this case, adduct **10** is thought to have formed intermediately, from which three possible structures **11**–**13** could be produced. Dihydrofuran **11** was formed by the attack of enolate (Path a), and isoxazoline *N*-oxide **12** is formed by the attack of nitronate derived from nitroketone (Path b). On the other hand, attack of the nitronate derived from nitrocinnamate affords isoxazoline *N*-oxide **13** (Path c).

When cinnamate **1b** was allowed to react with nitroketone **9** in the presence of triethylamine, the reaction mixture became somewhat complex, so only one cyclic product was isolated as a major product. Since a lot of small signals were observed in the ^1^H NMR spectrum of the reaction mixture, it is difficult to know whether cyclic products were formed as minor products or not. In the ^13^C NMR spectrum of the major product, a signal corresponding to carbonyl carbon was observed at 191 ppm, which indicated that the product had a ketone functionality; thus, the possibility of **11** was excluded. In the ^1^H-^1^H NOESY 2D NMR spectrum, a correlation was observed between the proton at the 5-position of the isoxazoline ring and the *ortho*-proton of the benzoyl group, by which the product was determined to be isoxazoline *N*-oxide **13**.

This result indicated that the nitronate ion substituted a nitro group via Path c. It is considered that the different reactivity of the two nitro groups was caused by the different electron-withdrawing ability of the ketone and the ester functionalities. The stronger electron-withdrawing toluoyl group increased the electrophilicity of the α-carbon and decreased the nucleophilicity of the nitronate ion, which facilitated the reaction via Path c leading to the predominant formation of **13**.

## 3. Experimental Section

### 3.1. General

All reagents were purchased from commercial sources and used without further purification. ^1^H and ^13^C NMR spectra were recorded on Bruker DPX-400 spectrometer (400 MHz and 100 MHz, respectively, Billerica, MA, USA) in CDCl_3_ using TMS as an internal standard. The ^13^C NMR assignments were performed via DEPT experiments. A Shimadzu IR spectrometer equipped with an ATR detector (Kyoto, Japan) was used to record infrared spectra. High-resolution mass spectra were obtained on an AB SCIEX Triplet TOF 4600 mass spectrometer (Framingham, MA, USA). Melting points were recorded on a Stanford Research Systems Optimelt automated melting point system (Sunnyvale, CA, USA) and were uncorrected.

### 3.2. Synthesis of Isoxazoline N-oxide ***4b***

To a solution of ethyl α-nitrocinnamate **1b** (94.6 mg, 0.43 mmol) in acetonitrile (1.3 mL), ethyl nitroacetate (48 μL, 0.43 mmol) and triethylamine (60 μL, 0.43 mmol) were added, and the resultant mixture was stirred at room temperature for 30 min. After removal of the solvent under reduced pressure, the residual brown oil was dissolved in ethyl acetate (10 mL) and washed with water (10 mL × 4), and then dried over magnesium sulfate. After removal of the solvent, the residue was purified by column chromatography on silica gel to afford isoxazoline *N*-oxide **4b** (eluted with hexane/EtOAc = 8/2, 121 mg, 0.41 mmol, 95%) as a yellow solid.

*3,5-Bis(ethoxycarbonyl)-4-phenyl-2-isoxazoline 2-oxide (***4b***)* [22]. Yellow solid, yield; 95%, m.p. 75–76 °C. ^1^H NMR (400 MHz, CDCl_3_) δ 7.40–7.30 (m, 5H), 4.93 (d, *J* = 2.8 Hz, 1H), 4.84 (d, *J* = 2.8 Hz, 1H), 4.35 (dq, *J* = 10.8, 7.2 Hz, 1H), 4.32 (dq, *J* = 10.8, 7.2 Hz, 1H), 4.21 (dq, *J* = 10.8, 7.2 Hz, 1H), 4.17 (dq, *J* = 10.8, 7.2 Hz, 1H), 1.35 (dd, *J* = 7.2, 7.2 Hz, 3H), 1.17 (dd, *J* = 7.2, 7.2 Hz, 3H); ^13^C NMR (100 MHz, CDCl3) δ 168.2 (C), 158.1 (C), 138.1 (C), 129.3 (CH), 128.7 (CH), 127.0 (CH), 109.0 (C), 78.8 (CH), 62.7 (CH2), 62.0 (CH2), 52.7 (CH), 14.1 (CH3), 13.9 (CH3).

### 3.3. Typical Procedure for Synthesis of 2,3-Dihydrofuran ***6***

To a solution of ethyl α-nitrocinnamate **1b** (71.5 mg, 0.32 mmol) in acetonitrile (1.0 mL), ethyl acetoacetate **5a** (41 μL, 0.32 mmol) and triethylamine (45 μL, 0.32 mmol) were added, and the resultant mixture was heated at 60 °C for 3 h. After removal of the solvent under reduced pressure, the residual orange oil was purified by column chromatography on silica gel to afford 2,3-dihydrofuran **6a** (eluted with hexane/EtOAc = 1/1, 88 mg, 0.29 mmol, 92%) as a pale yellow oil. When other 1,3-dicarbonyl compounds **5** were used, the experiment was conducted in the same way.

*2,4-Bis(ethoxycarbonyl)-2,3-dihydro-5-methyl-3-phenylfuran* (**6a**) [23]. Pale yellow oil, yield; 92%. ^1^H NMR (400 MHz, CDCl_3_) δ 7.34–7.30 (m, 2H), 7.27–7.22 (m, 3H), 4.83 (d, *J* = 4.8 Hz, 1H), 4.41 (dq, *J* = 4.8, 1.2 Hz, 1H), 4.30 (dq, *J* = 10.8, 7.2 Hz, 1H), 4.26 (dq, *J* = 10.8, 7.2 Hz, 1H), 4.04 (dq, *J* = 10.8, 7.2 Hz, 1H), 3.98 (dq, *J* = 10.8, 7.2 Hz, 1H), 2.40 (d, *J* = 1.2 Hz, 3H), 1.32 (dd, *J* = 7.2, 7.2 Hz, 3H), 1.07 (dd, *J* = 7.2, 7.2 Hz, 3H); ^13^C NMR (100 MHz, CDCl_3_) δ 170.1 (C), 168.4 (C), 164.9 (C), 142.6 (C), 128.6 (CH), 127.2 (CH), 127.1(CH), 106.4 (C), 85.8 (CH), 61.8 (CH_2_), 59.6 (CH_2_), 52.8 (CH), 14.2 (CH_3_), 14.1 (CH_3_), 14.1 (CH_3_); IR (ATR/cm^−1^) 1755, 1701, 1651, 1207, 1088, 1038; HRMS (ESI/TOF) calcd. for [M + H]^+^ C_17_H_21_O_5_: 305.1384, found: 305.1384.

*2,4-Bis(ethoxycarbonyl)-5-trifluoromethyl-2,3-dihydro-3-phenylfuran* (**6b**). Yellow oil, yield; 43%. ^1^H NMR (400 MHz, CDCl_3_) δ 7.4–7.2 (m, 5H), 4.99 (d, *J* = 4.8 Hz, 1H), 4.62 (dq, *J* = 4.8, 2.4 Hz, 1H), 4.32 (dq, *J* = 11.6, 7.2 Hz, 1H), 4.30 (dq, *J* = 11.6, 7.2 Hz, 1H), 4.12 (dq, *J* = 10.8, 7.2 Hz, 1H), 4.05 (dq, *J* = 10.8, 7.2 Hz, 1H), 1.33 (dd, *J* = 7.2, 7.2 Hz, 3H), 1.13 (dd, *J* = 7.2, 7.2 Hz, 3H); ^13^C NMR (100 MHz, CDCl_3_) δ 168.5 (C), 161.2 (C), 151.2 (C, q, *J* = 40.0 Hz), 139.8 (C), 129.1 (CH), 128.1 (CH), 127.2 (CH), 118.0 (C, q, *J* = 271.0 Hz), 113.0 (C, q, *J* = 3.0 Hz), 86.2 (CH), 62.3 (CH_2_), 61.1 (CH_2_), 53.8 (CH), 14.1 (CH_3_), 13.7 (CH_3_); IR (ATR/cm^−1^) 1759, 1728, 1200, 1157, 1111; HRMS (ESI/TOF) calcd. for [M + H]^+^ C_17_H_18_F_3_O_5_: 359.1101, found: 359.1092.

*2,4-Bis(ethoxycarbonyl)-2,3-dihydro-1,3-diphenylfuran* (**6c**). Colorless oil, yield; 62%. ^1^H NMR (400 MHz, CDCl_3_) δ 7.98–7.95 (m, 2H), 7.48–7.42 (m, 3H), 7.42–7.35 (m, 4H), 7.35–7.28 (m, 1H), 4.96 (d, *J* = 4.4 Hz, 1H), 4.62 (d, *J* = 4.4 Hz, 1H), 4.34 (dq, *J* = 10.8, 7.2 Hz, 1H), 4.31 (dq, *J* = 10.8, 7.2 Hz, 1H), 4.01 (dq, *J* = 10.8, 7.2 Hz, 1H), 3.98 (dq, *J* = 10.8, 7.2 Hz, 1H), 1.36 (dd, *J* = 7.2, 7.2 Hz, 3H), 1.03 (dd, *J* = 7.2, 7.2 Hz, 3H); ^13^C NMR (100 MHz, CDCl_3_) δ 170.2 (C), 165.4 (C), 164.1 (C), 142.5 (C), 130.1 (CH), 129.8 (CH), 129.2 (C), 128.8 (CH), 127.7 (CH), 127.4 (CH), 127.2 (CH), 106.7 (C), 85.0 (CH), 61.8 (CH_2_), 59.9 (CH_2_), 54.1 (CH), 14.2 (CH_3_), 13.9 (CH_3_); IR (ATR/cm^−1^) 1751, 1697, 1628, 1203, 1076, 752, 694; HRMS (ESI/TOF) calcd. for [M + H]^+^ C_22_H_23_O_5_: 367.1540, found: 367.1540.

*4-Ethanoyl-2-ethoxycarbonyl-2,3-dihydro-5-methyl-3-phenylfuran* (**6d**) [24]. Yellow solid, yield; quant., m.p. 63–64 °C. ^1^H NMR (400 MHz, CDCl_3_) δ 7.37–7.33 (m, 2H), 7.30–7.23 (m, 3H), 4.72 (d, *J* = 4.8 Hz, 1H), 4.49 (dq, *J* = 4.8, 1.2 Hz, 1H), 4.31 (dq, *J* = 10.8, 7.2 Hz, 1H), 4.27 (dq, *J* = 10.8, 7.2 Hz, 1H), 2.44 (d, *J* = 1.2 Hz, 3H), 1.95 (s, 3H), 1.34 (dd, *J* = 7.2, 7.2 Hz, 3H); ^13^C NMR (100 MHz, CDCl_3_) δ 194.3 (C), 170.0 (C), 168.6 (C), 142.2 (C), 129.1 (CH), 127.6 (CH), 127.2 (CH), 115.1 (C), 86.0 (CH), 61.9 (CH_2_), 53.3 (CH), 29.6 (CH_3_), 14.9 (CH_3_), 14.2 (CH_3_); IR (ATR/cm^−1^) 1755, 1674, 1624, 1604, 1196, 1038; HRMS (ESI/TOF) calcd. for [M + H]^+^ C_16_H_18_O_4_Na: 297.1097, found: 297.1099.

*5,6-Cyclohexa-2-ethoxycarbonyl-2,3-dihydro-3-phenylfuran-4-one* (**6e**) [25]. Yellow oil, yield; quant. ^1^H NMR (400 MHz, CDCl_3_) δ 7.34–7.31 (m, 2H), 7.27–7.21 (m, 3H), 4.96 (d, *J* = 4.8 Hz, 1H), 4.46 (br d, *J* = 4.8 Hz, 1H), 4.32 (dq, *J* = 10.8, 7.2 Hz, 1H), 4.27 (dq, *J* = 10.8, 7.2 Hz, 1H), 2.68–2.65 (m, 2H), 2.44–2.31 (m, 2H), 2.19–2.10 (m, 2H), 1.33 (dd, *J* = 7.2, 7.2 Hz, 3H); ^13^C NMR (100 MHz, CDCl_3_) δ 194.3 (C), 177.4 (C), 169.5 (C), 141.1 (C), 128.9 (CH), 127.4 (CH), 127.0 (CH), 115.8 (C), 88.0 (CH), 62.0 (CH_2_), 49.8 (CH), 36.8 (CH_2_), 23.9 (CH_2_), 21.7 (CH_2_), 14.2 (CH_3_); IR (ATR/cm^−1^) 1751, 1639, 1396, 1219, 748; HRMS (ESI/TOF) calcd. for [M + H]^+^ C_17_H_19_O_4_: 287.1278, found: 287.1278.

*3-Ethoxycarbonyl-4,5-dihydro-5-(4-methylbenzoyl)-4-phenylisoxazoline 2-oxide* (**13**). Yellow oil, yield; 64%. ^1^H NMR (400 MHz, CDCl_3_) δ 7.81 (d, *J* = 8.0 Hz, 2H), 7.42–7.36 (m, 5H), 7.29 (d, *J* = 8.0 Hz, 2H), 5.66 (d, *J* = 3.6 Hz, 1H), 5.12 (d, *J* = 3.6 Hz, 1H), 4.19 (dq, *J* = 10.8, 7.2 Hz, 1H), 4.14 (dq, *J* = 10.8, 7.2 Hz, 1H), 2.43 (s, 3H), 1.13 (dd, *J* = 7.2, 7.2 Hz, 3H); ^13^C NMR (100 MHz, CDCl_3_) δ 191.3 (C), 158.4 (C), 146.0 (C), 138.6 (C), 130.9 (C), 129.9 (CH), 129.5 (CH), 129.5 (CH), 128.8 (CH), 127.6 (CH), 109.8 (C), 81.7 (CH), 62.0 (CH_2_), 51.8 (CH), 22.0 (CH_3_), 14.0 (CH_3_); IR (KBr/cm^−1^) 1736, 1697, 1628, 1606, 1228, 740; HRMS (ESI/TOF) calcd. for [M + H]^+^ C_20_H_20_NO_5_: 354.1336 found: 354.1337.

## 4. Conclusions

2,3-Dihydrofurans and isoxazoline *N*-oxides were synthesized from α-nitrocinnamate **1** and active methylene compounds by conjugate addition and the subsequent *O*-attack. Via a series of reactions using several substrates, the nitro group increased the electrophilicity of the α-carbon and served as a good leaving group. The nitro group also served as a good nucleophile when it was converted to nitronate ion, which is more reactive than the enolate ion of ketone or ester functionalities. These results will be useful information for researchers studying synthetic chemistry using the multi-functionalities of a nitro group.

## Figures and Tables

**Table 1:**
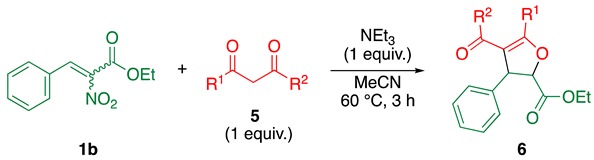
Synthesis of functionalized 2,3-dihydrofuran **6**.

Entry	R^1^	R^2^	5 and 6	Yield/%
1	Me	OEt	**a**	92
2	CF_3_	OEt	**b**	43
3	Ph	OEt	**c**	62
4	Me	Me	**d**	quant.
5	–(CH_2_)_3_–		**e**	quant.
6	OEt	OEt	**f**	0

## Supplementary Materials

The following are available online. ^1^H and ^13^C NMR spectra of **2a**, **4**, **6** and **13**.
