# Supplementary material for: Comparison of Substituting Ability of Nitronate versus Enolate for Direct Substitution of a Nitro Group
Source: Molecules. 2020 Apr 28;25(9):2048. doi: 10.3390/molecules25092048 (PMC7248694; doi:10.3390/molecules25092048)
Supplement: Supplementary file 1 [file molecules-25-02048-s001.pdf]

Comparison of Substituting Ability of Nitronate versus Enolate for Direct Substitution of a Nitro Group

Yusuke Mukaijo [1], Soichi Yokoyama [1,2,3] and Nagatoshi Nishiwaki [1,2,*]

1 School of Environmental Science and Engineering, Kochi University of Technology, Tosayamada, Kami, Kochi 782-8502, Japan
2 Research Center for Molecular Design, Kochi University of Technology, Tosayamada, Kami, Kochi 782-8502, Japan
3 The Institute of Scientific and Industrial Research, Osaka University, Mihogaoka, Ibaraki, Osaka 567-0047, Japan
* Correspondence: nishiwaki.nagatoshi@kochi-tech.ac.jp; Tel.: +81-887-57-2517

Supporting Information

Table of Contents

Compound	Spectra	Page
Reaction mixture including adduct **2a** and *N*-propylimine **3**	^{1}H NMR	S2
3,5-Bis(methoxycarbonyl)-4-phenyl-2-isoxazoline 2-oxide (**4a**)	^{1}H and ^{13}C NMR	S3–S4
3,5-Bis(ethoxycarbonyl)-4-phenyl-2-isoxazoline 2-oxide (**4b**)	^{1}H and ^{13}C NMR	S5–S6
2,4-Bis(ethoxycarbonyl)-2,3-dihydro-5-methyl-3-phenylfuran (**6a**)	^{1}H and ^{13}C NMR	S7–S8
2,4-Bis(ethoxycarbonyl)-5-trifluoromethyl-2,3-dihydro-3-phenylfuran (**6b**)	^{1}H and ^{13}C NMR	S9–S10
2,4-Bis(ethoxycarbonyl)-2,3-dihydro-1,3-diphenylfuran (**6c**)	^{1}H and ^{13}C NMR	S11–S12
4-Ethanoyl-2-ethoxycarbonyl-2,3-dihydro-5-methyl-3-phenylfuran (**6d**)	^{1}H and ^{13}C NMR	S13–S14
5,6-Cyclohexa-2-ethoxycarbonyl-2,3-dihydro-3-phenylfuran-4-one (**6e**)	^{1}H and ^{13}C NMR	S15–S16
3-Ethoxycarbonyl-4,5-dihydro-5-(4-methylbenzoyl)-4-phenylisoxazoline 2-oxide (**13**)	^{1}H, ^{13}C and ^{1}H-^{1}H COSY 2D NMR	S17–S19

^{1}H NMR (400 MHz, $CDCl_3$) of the reaction mixture including adduct **2a** (blue mark) and *N*-propylimine **3** (red mark)

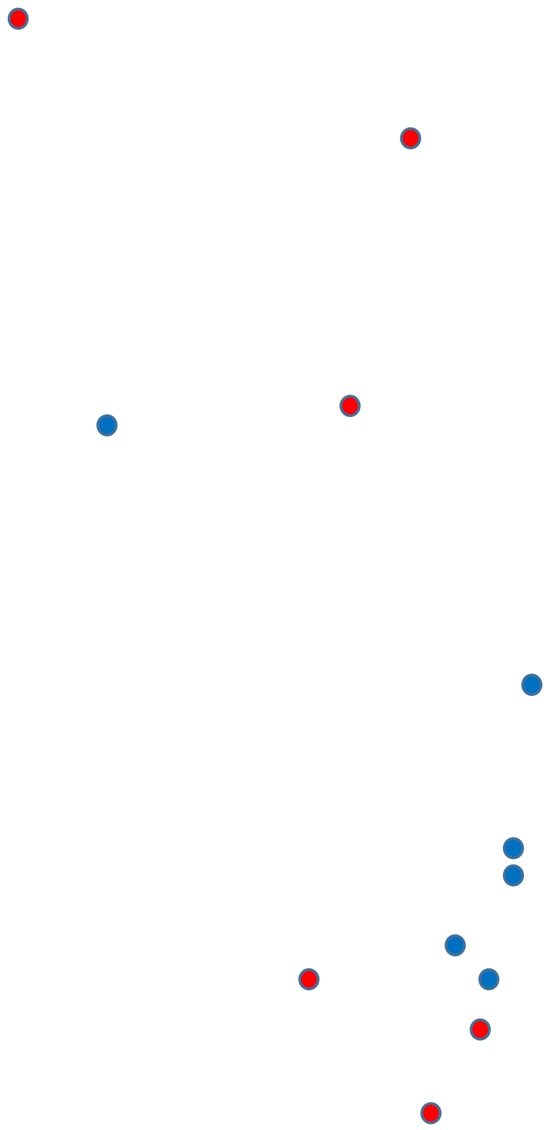

3,5-Bis(methoxycarbonyl)-4-phenyl-2-isoxazoline 2-oxide (**4a**)

^{1}H NMR (400 MHz, $CDCl_3$)

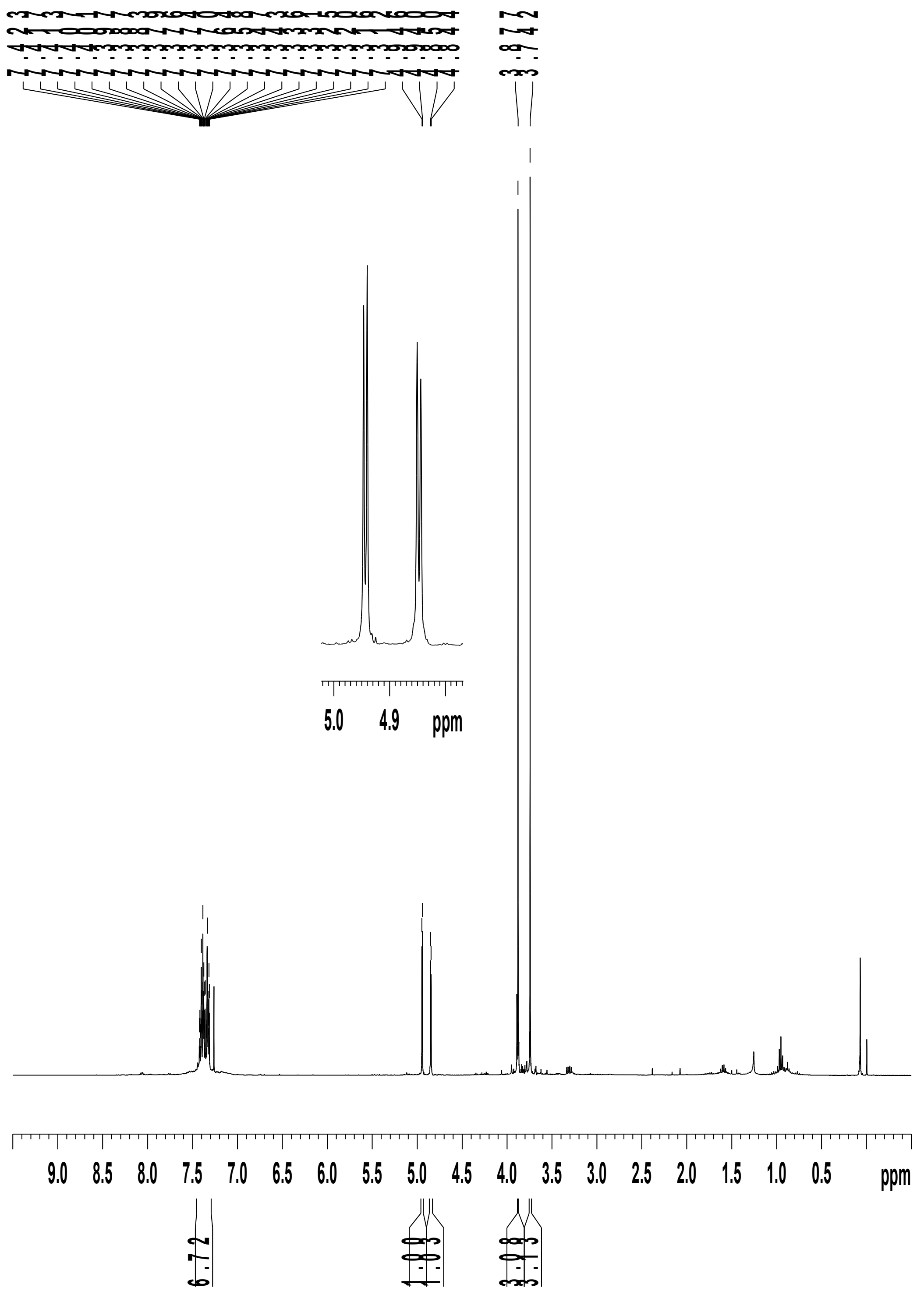

^{13}C NMR (100 MHz, $CDCl_3$)

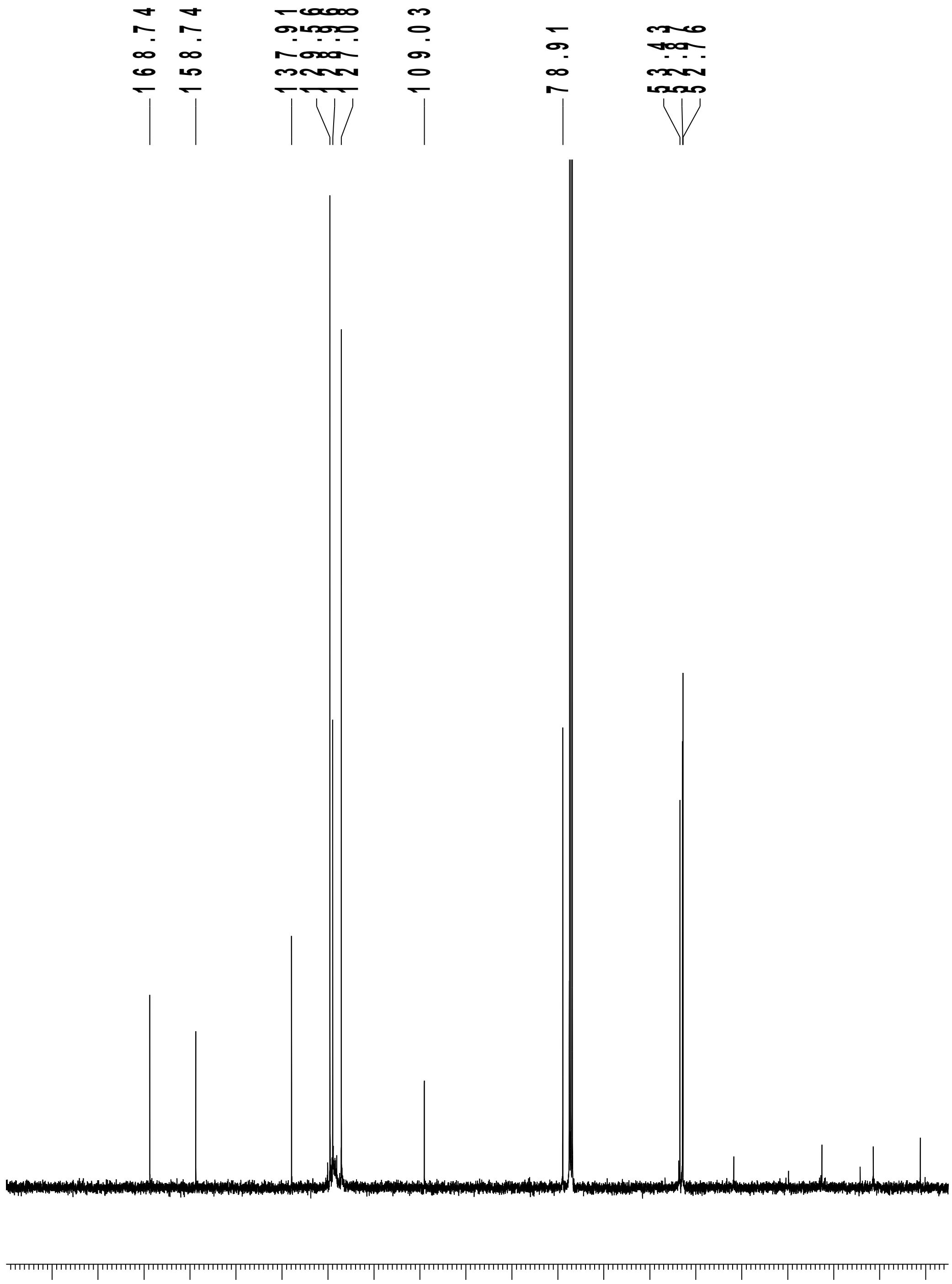

3,5-Bis(ethoxycarbonyl)-4-phenyl-2-isoxazoline 2-oxide (**4b**)

^{1}H NMR (400 MHz, $CDCl_3$)

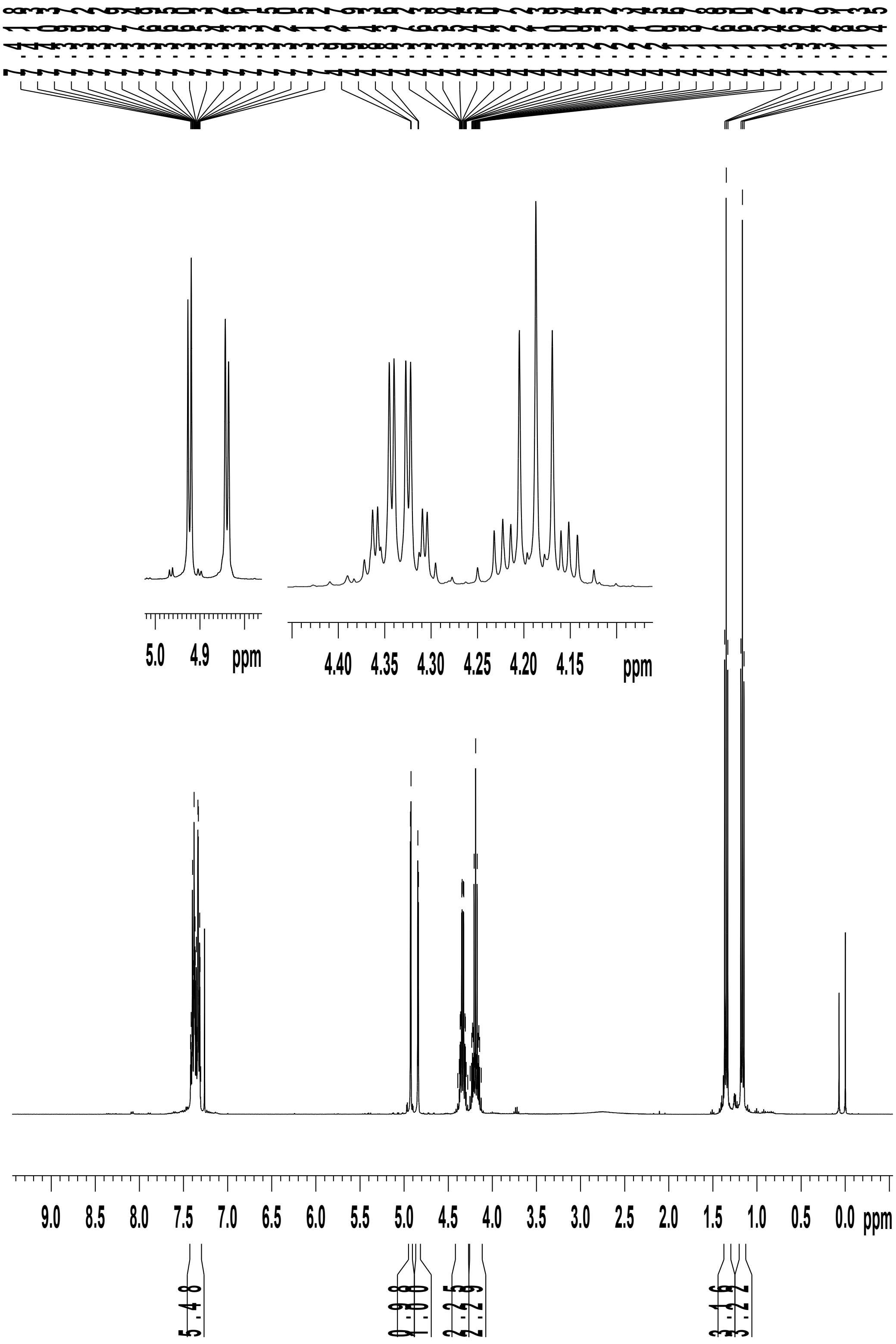

^{13}C NMR (100 MHz, $CDCl_3$)

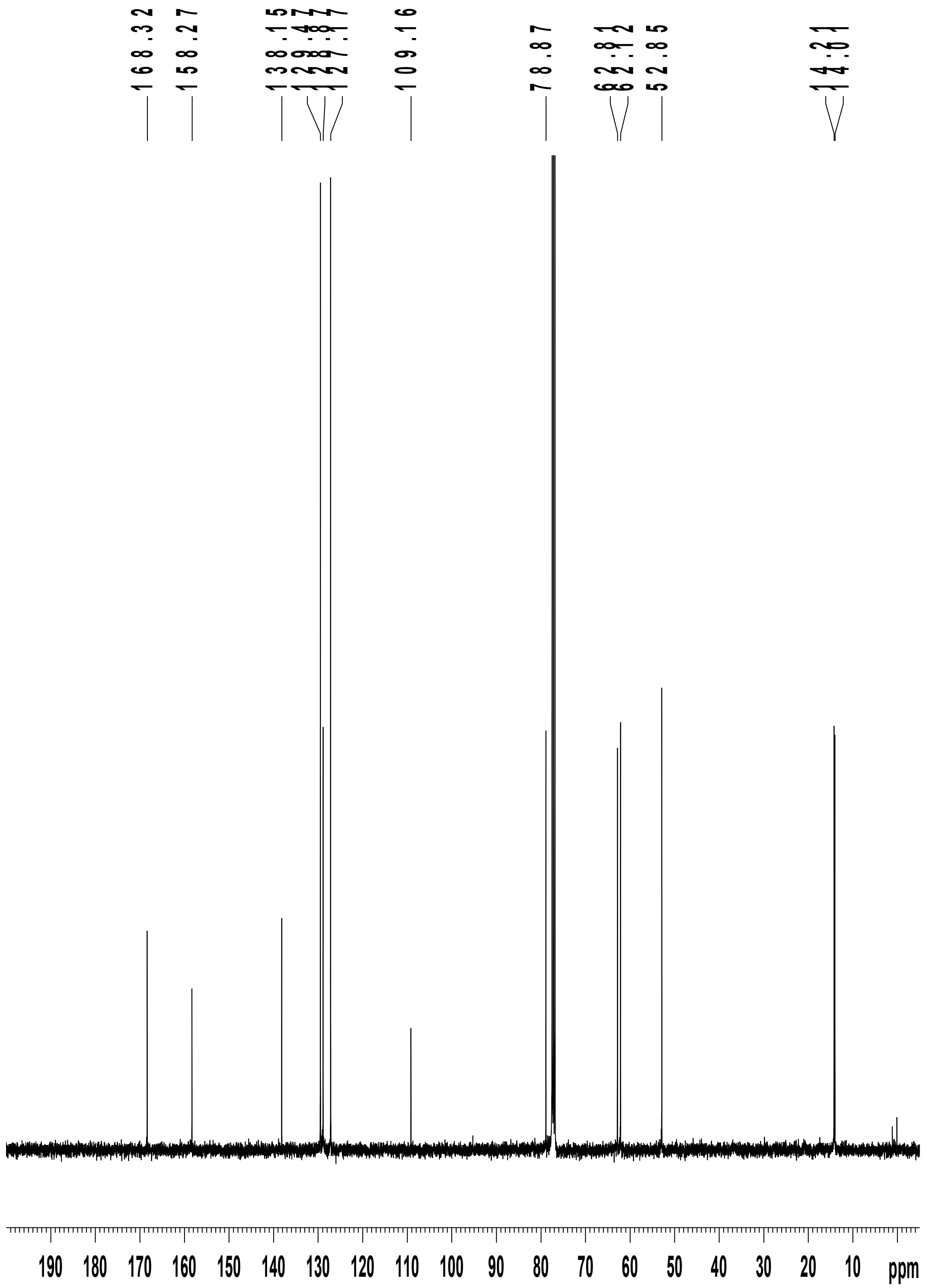

2,4-Bis(ethoxycarbonyl)-2,3-dihydro-5-methyl-3-phenylfuran (**6a**)

^{1}H NMR (400 MHz, $CDCl_3$)

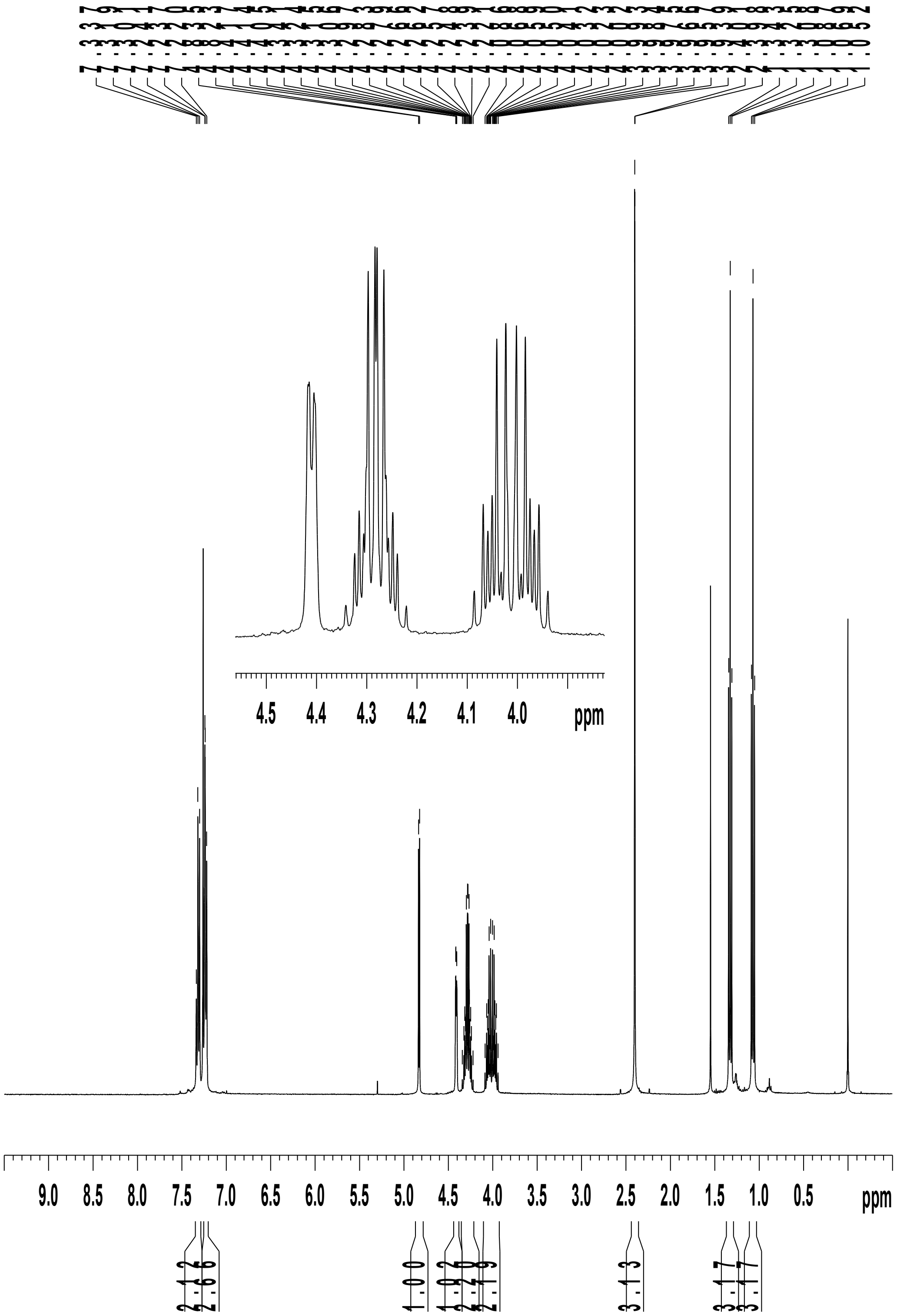

^{13}C NMR (100 MHz, $CDCl_3$)

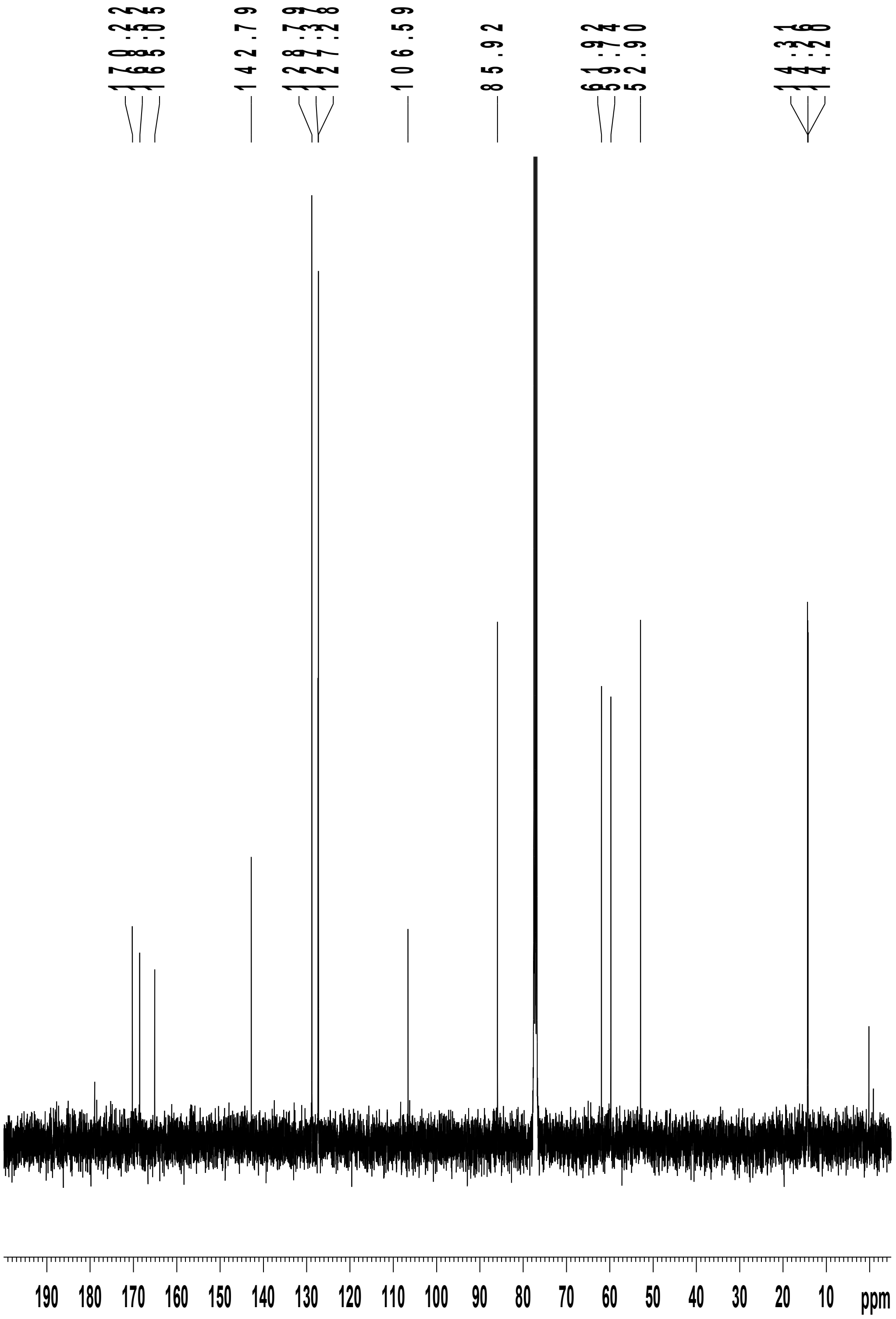

2,4-Bis(ethoxycarbonyl)-5-trifluoromethyl-2,3-dihydro-3-phenylfuran (**6b**)

^{1}H NMR (400 MHz, $CDCl_3$)

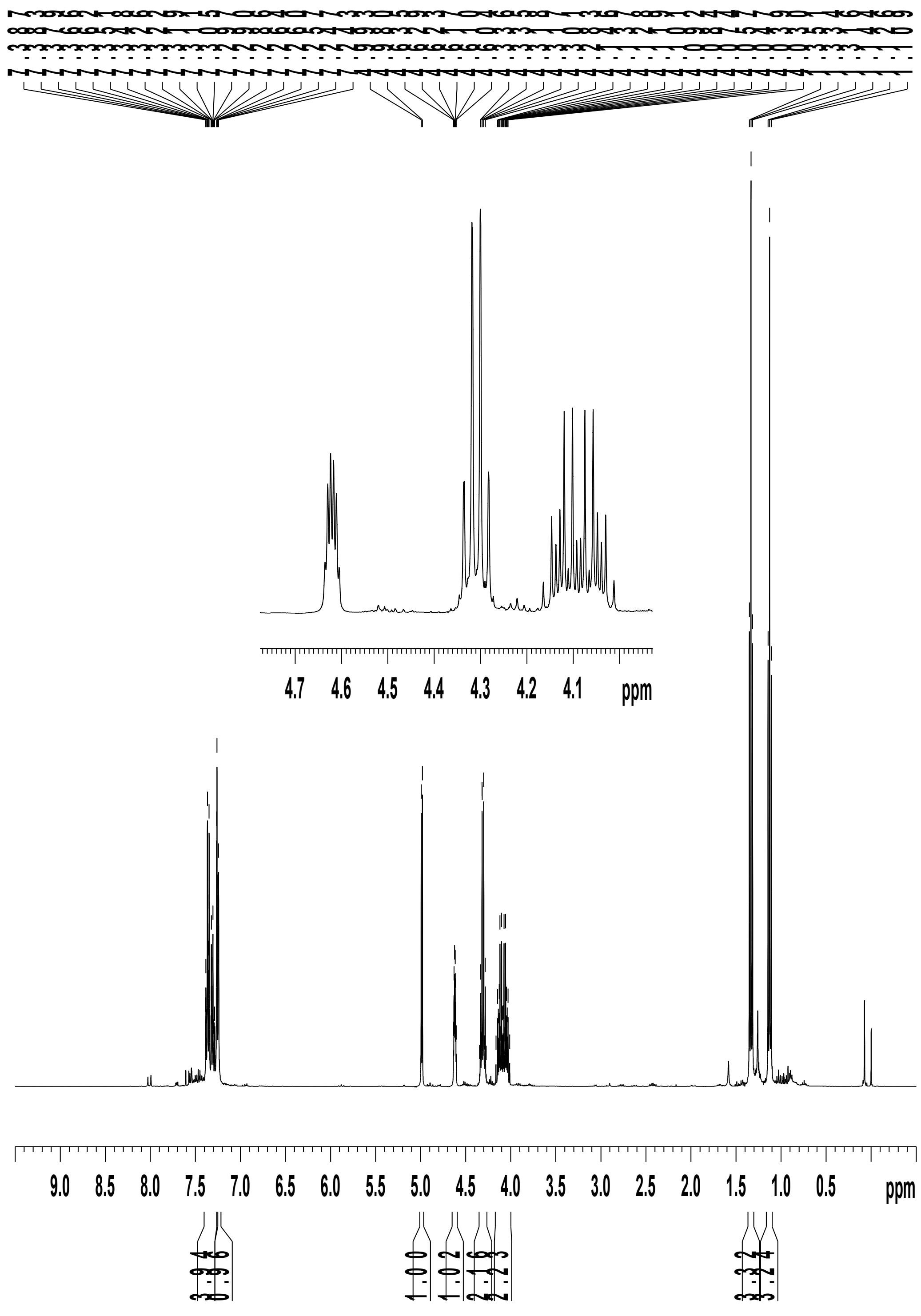

^{13}C NMR (100 MHz, $CDCl_3$)

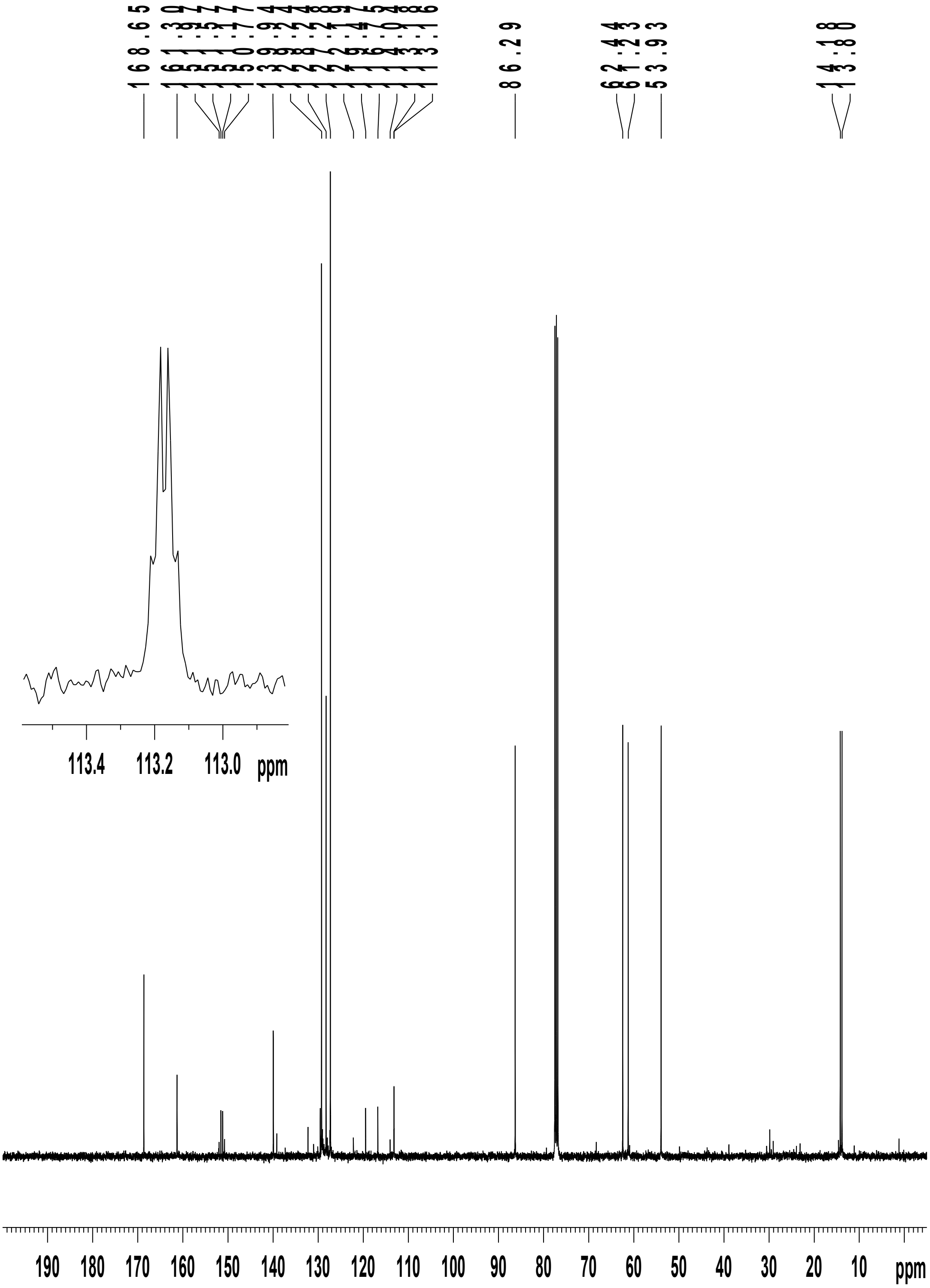

2,4-Bis(ethoxycarbonyl)-2,3-dihydro-1,3-diphenylfuran (**6c**)

^{1}H NMR (400 MHz, $CDCl_3$)

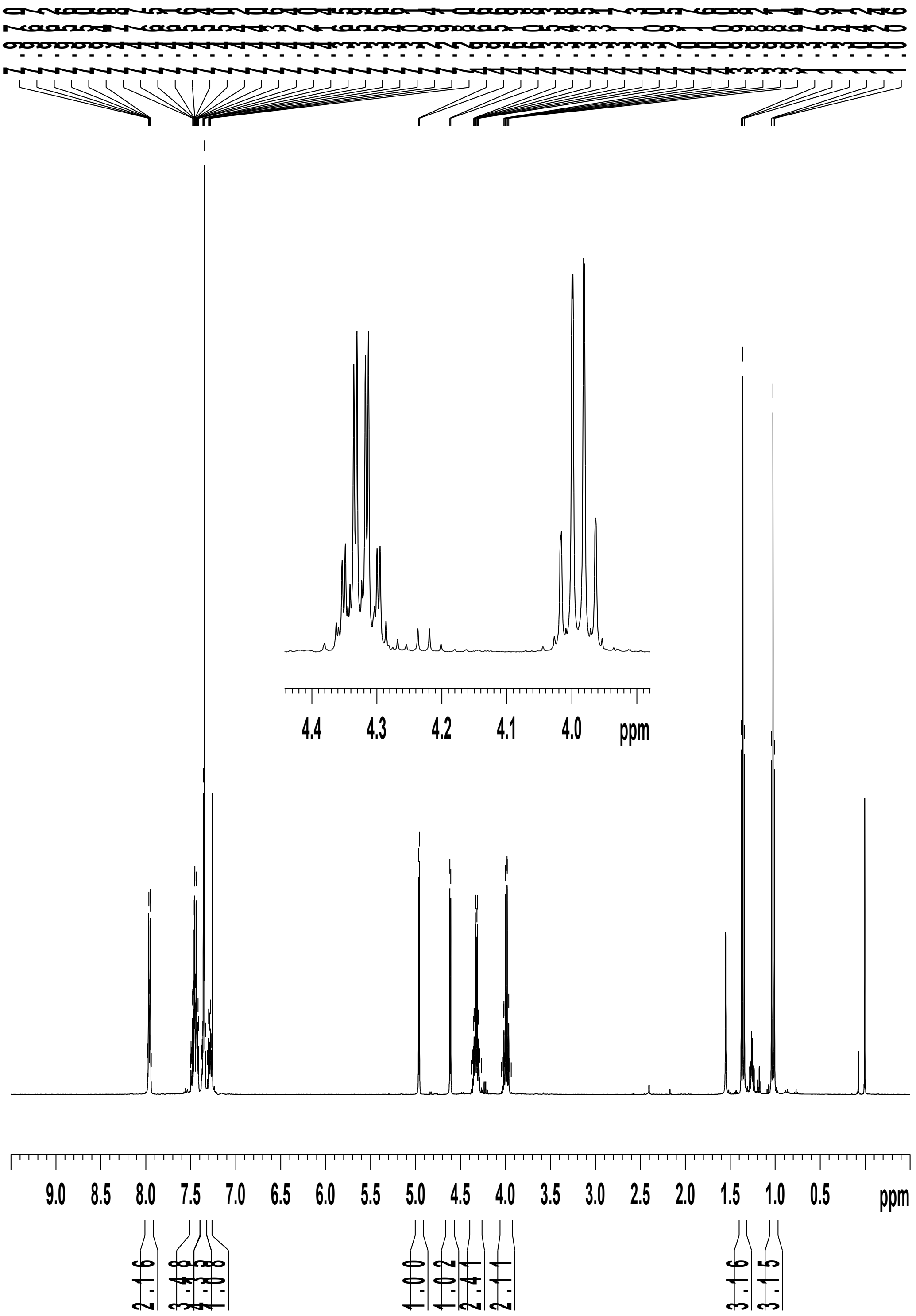

^{13}C NMR (100 MHz, $CDCl_3$)

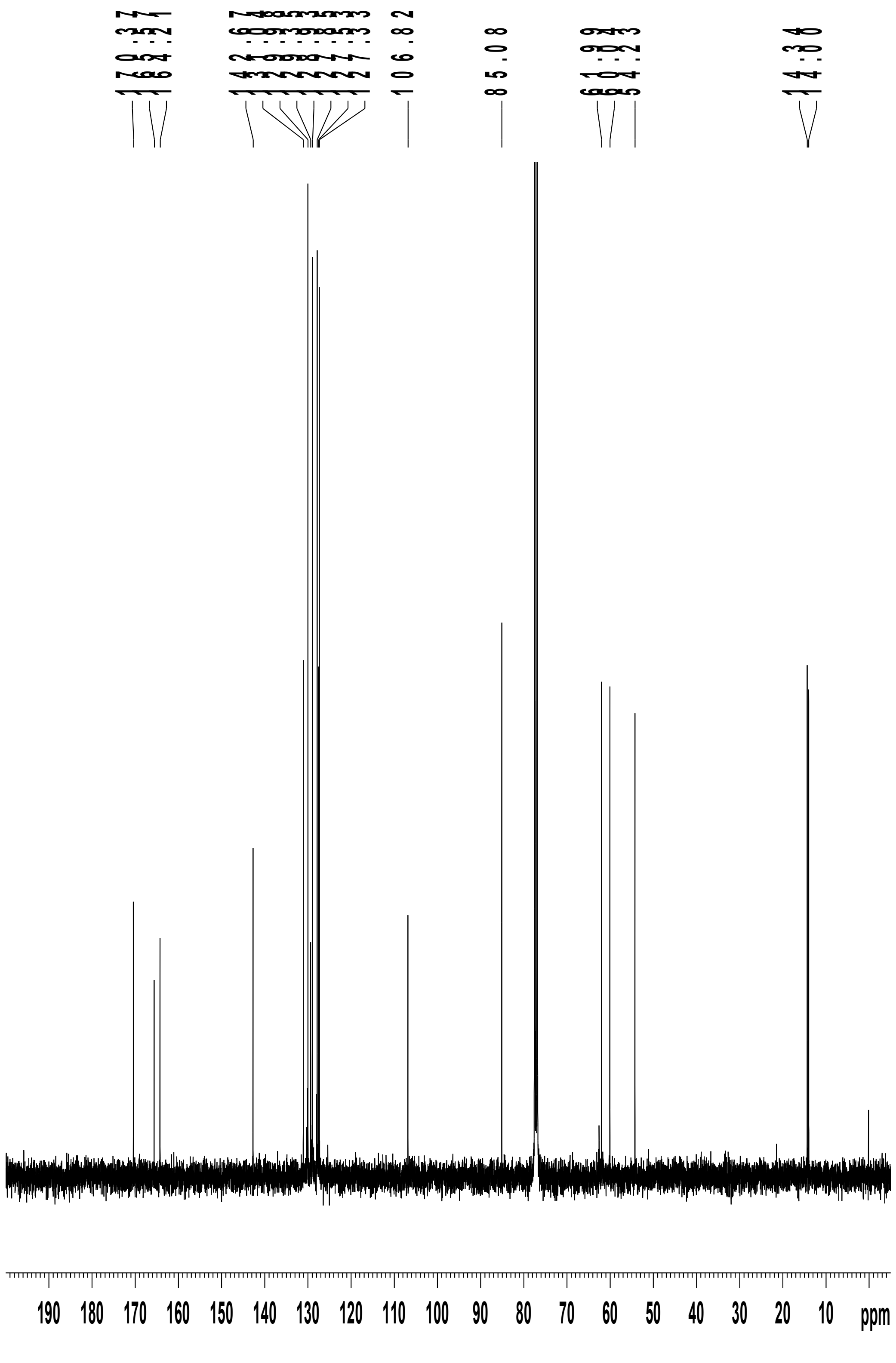

4-Ethanoyl-2-ethoxycarbonyl-2,3-dihydro-5-methyl-3-phenylfuran (**6d**)

^{1}H NMR (400 MHz, $CDCl_3$)

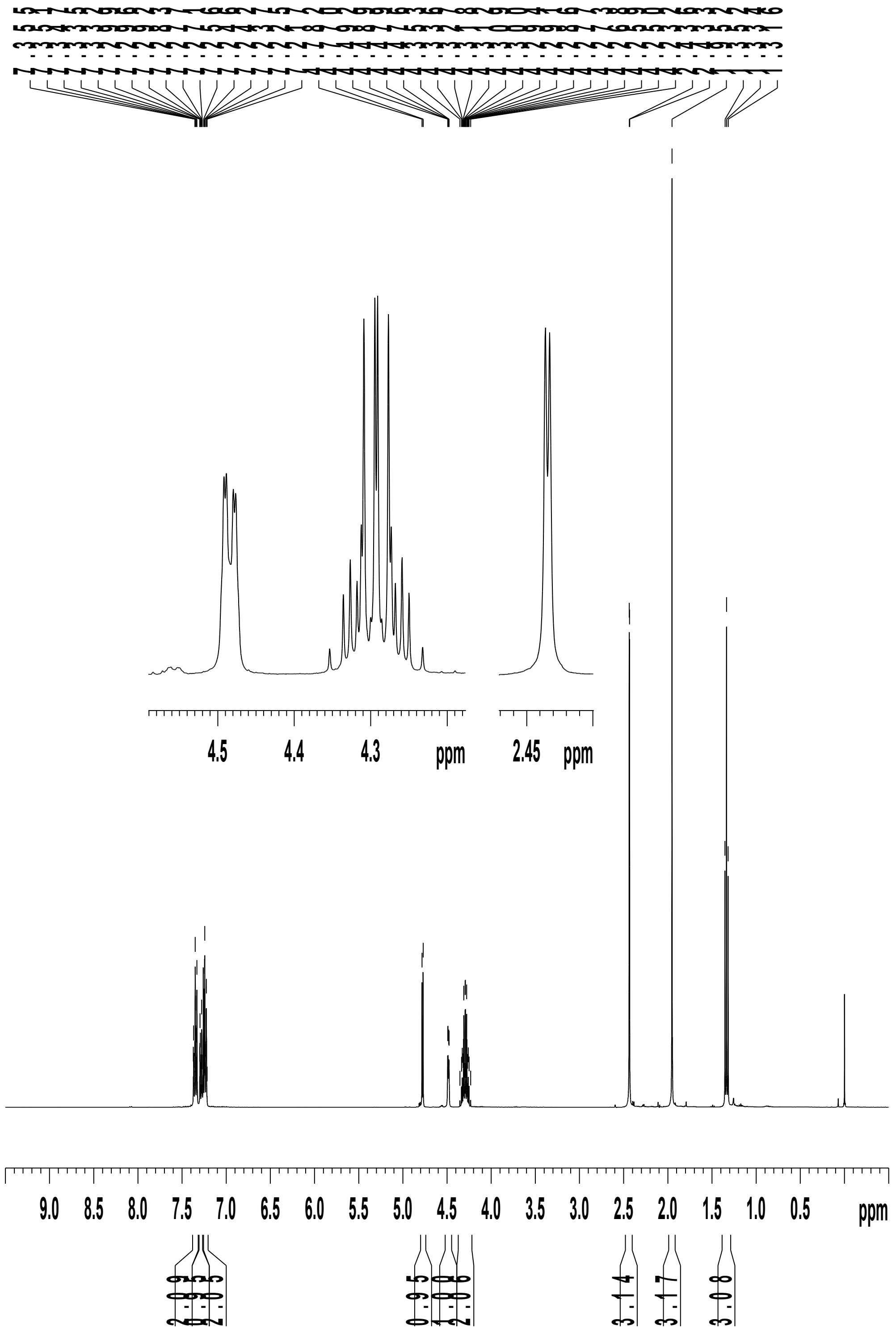

^{13}C NMR (100 MHz, $CDCl_3$)

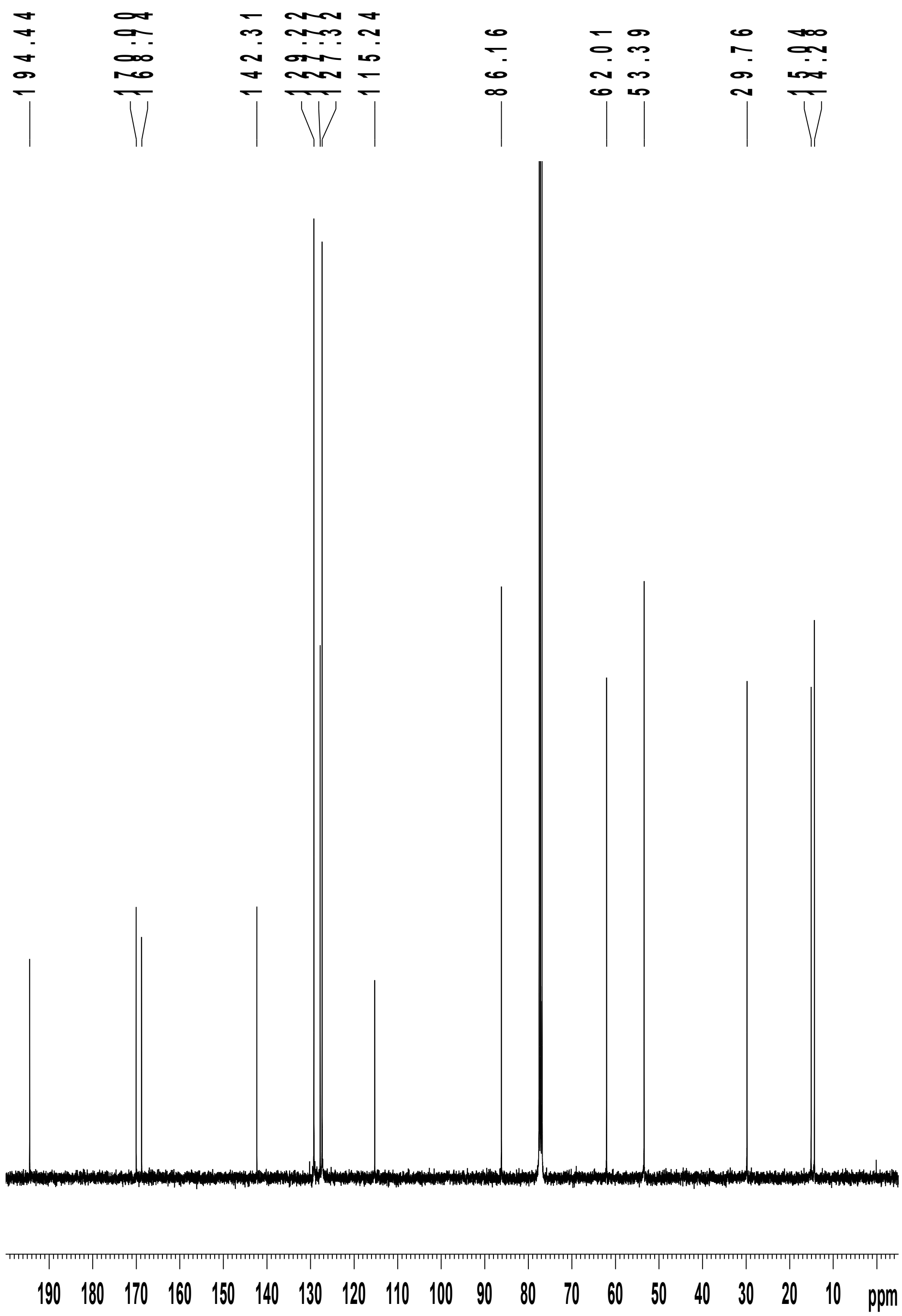

5,6-Cyclohexa-2-ethoxycarbonyl-2,3-dihydro-3-phenylfuran-4-one (**6e**)

^{1}H NMR (400 MHz, $CDCl_3$)

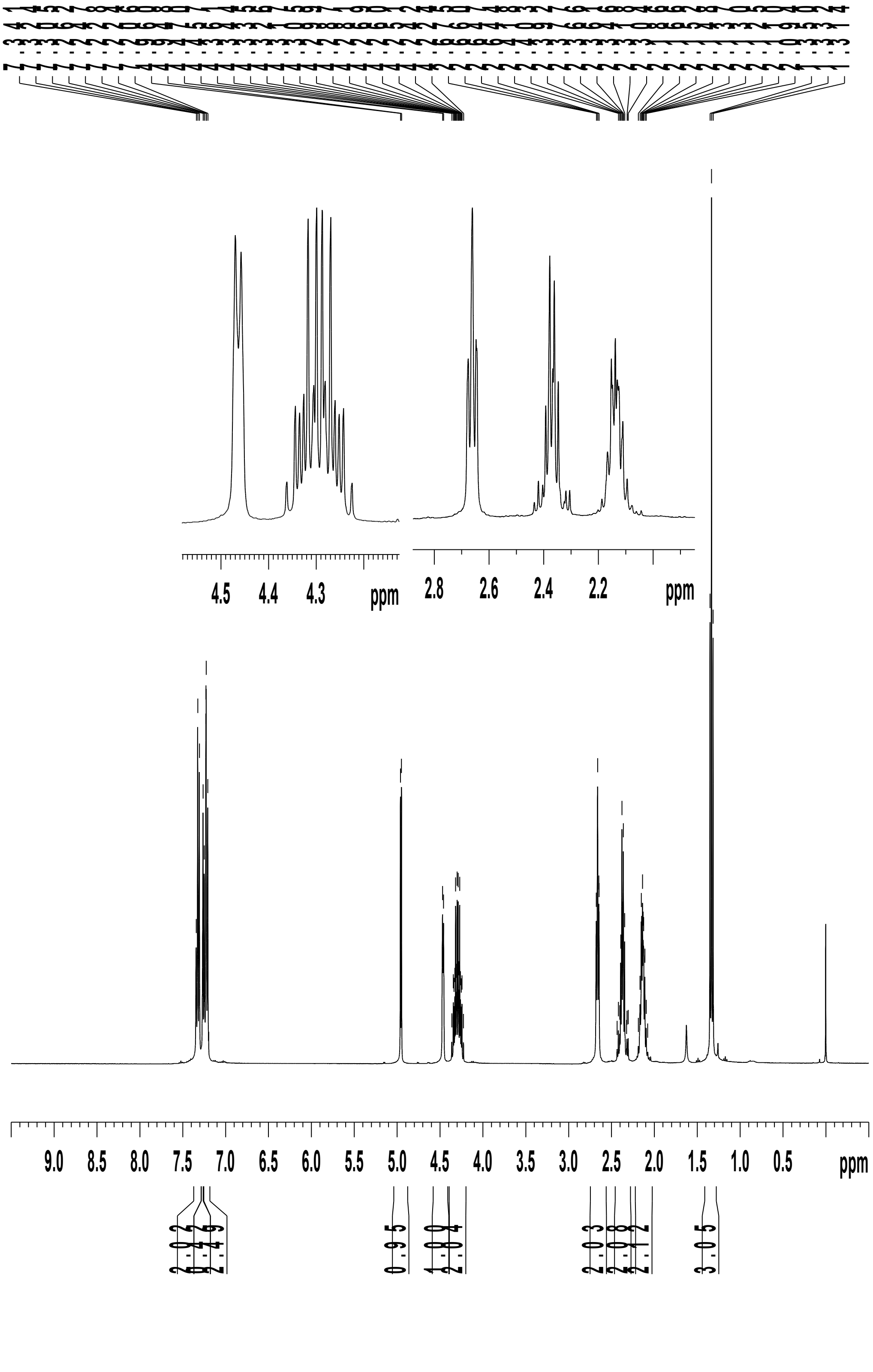

^{13}C NMR (100 MHz, $CDCl_3$)

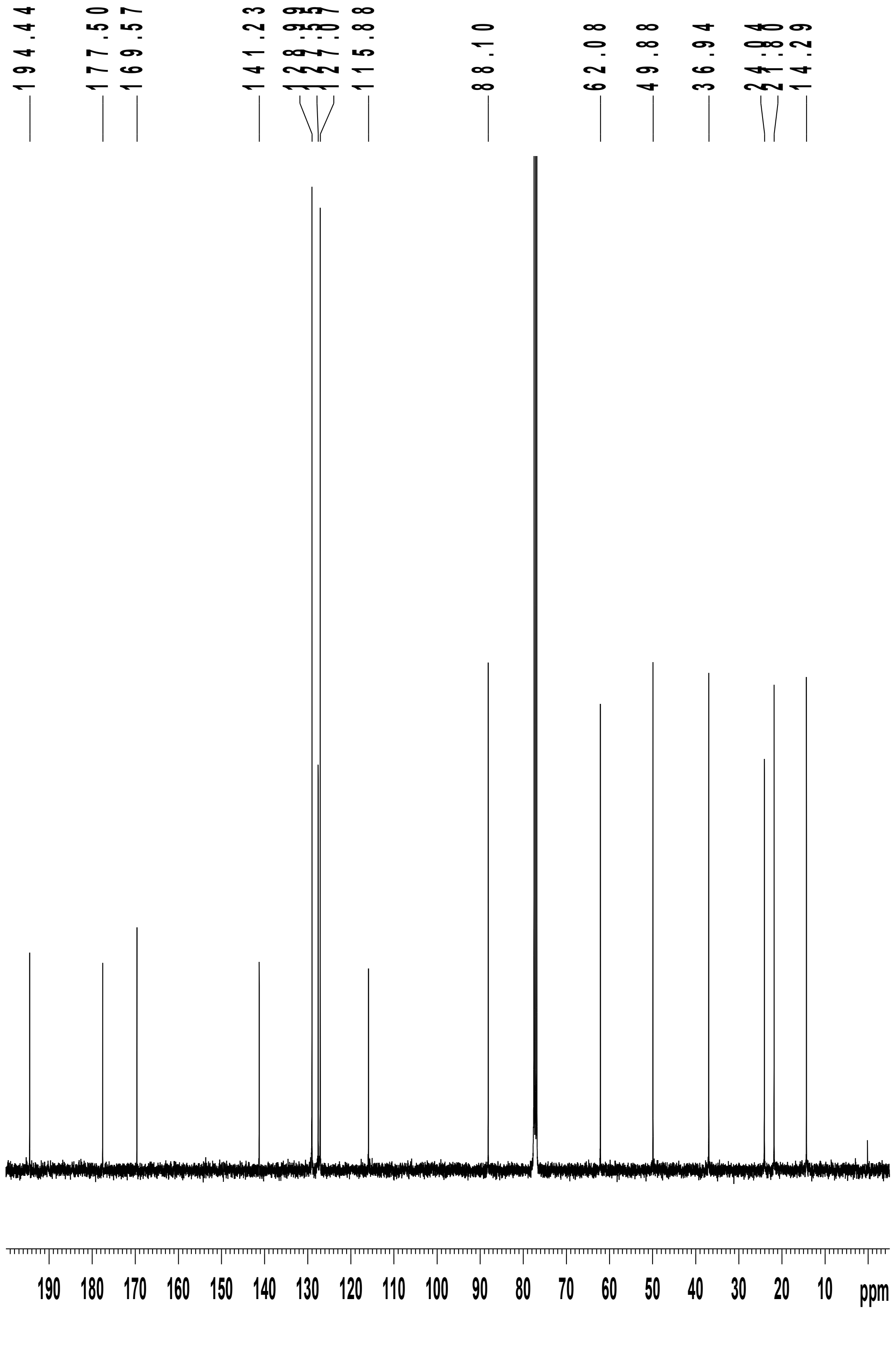

3-Ethoxycarbonyl-4,5-dihydro-5-(4-methylbenzoyl)-4-phenylisoxazoline 2-oxide (**13**)
^{1}H NMR (400 MHz, $CDCl_3$)

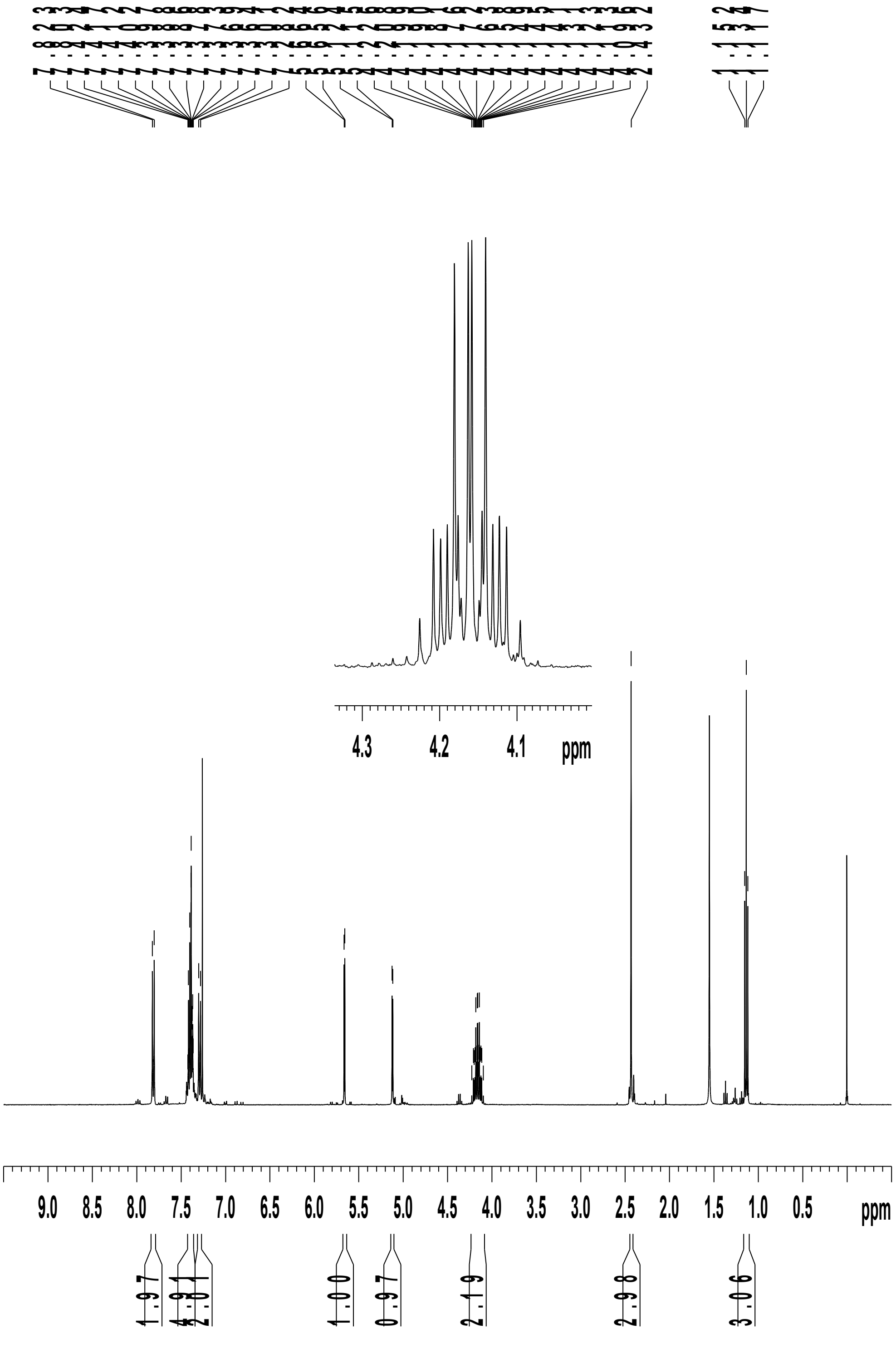

^{13}C NMR (100 MHz, $CDCl_3$)

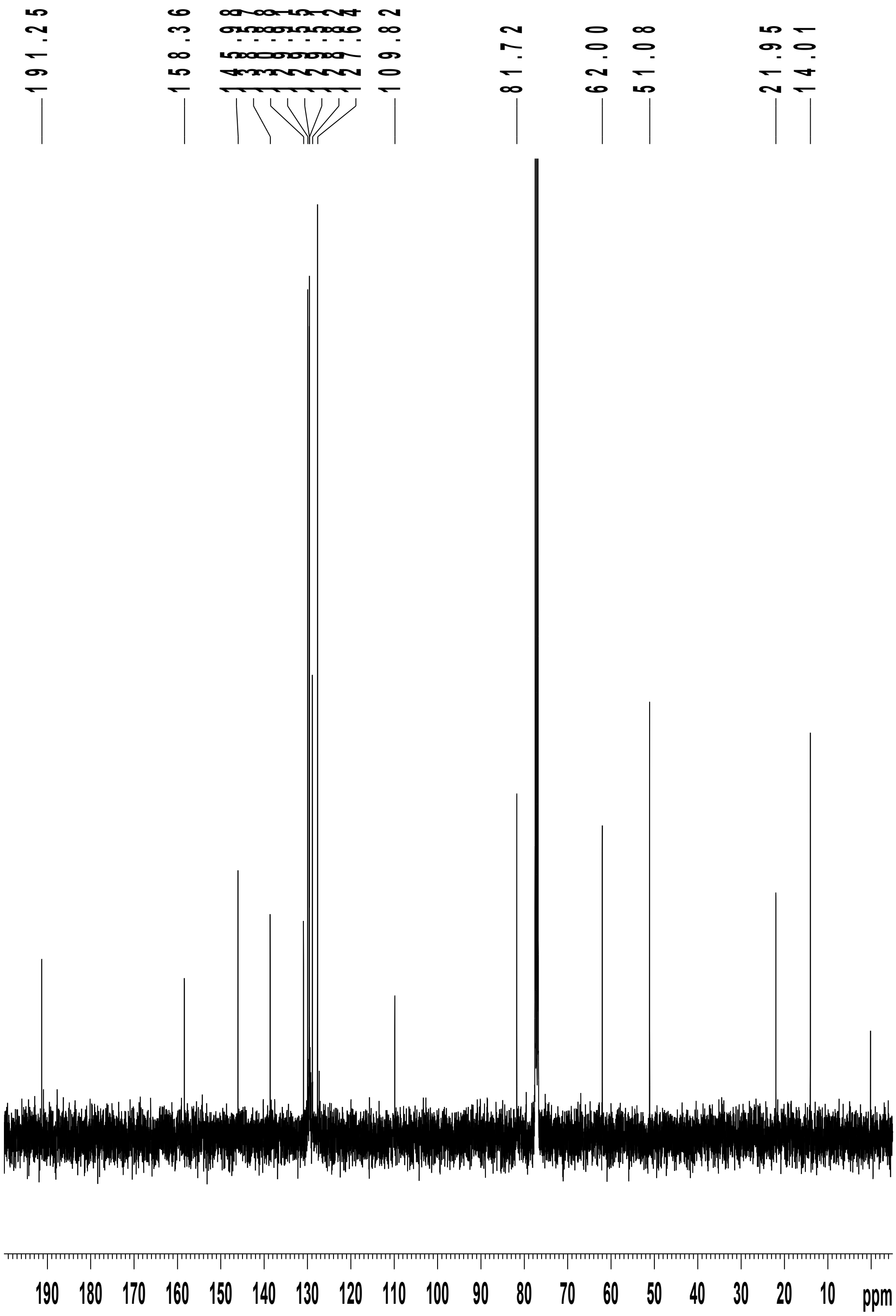

^1H-^1H 2D NMR

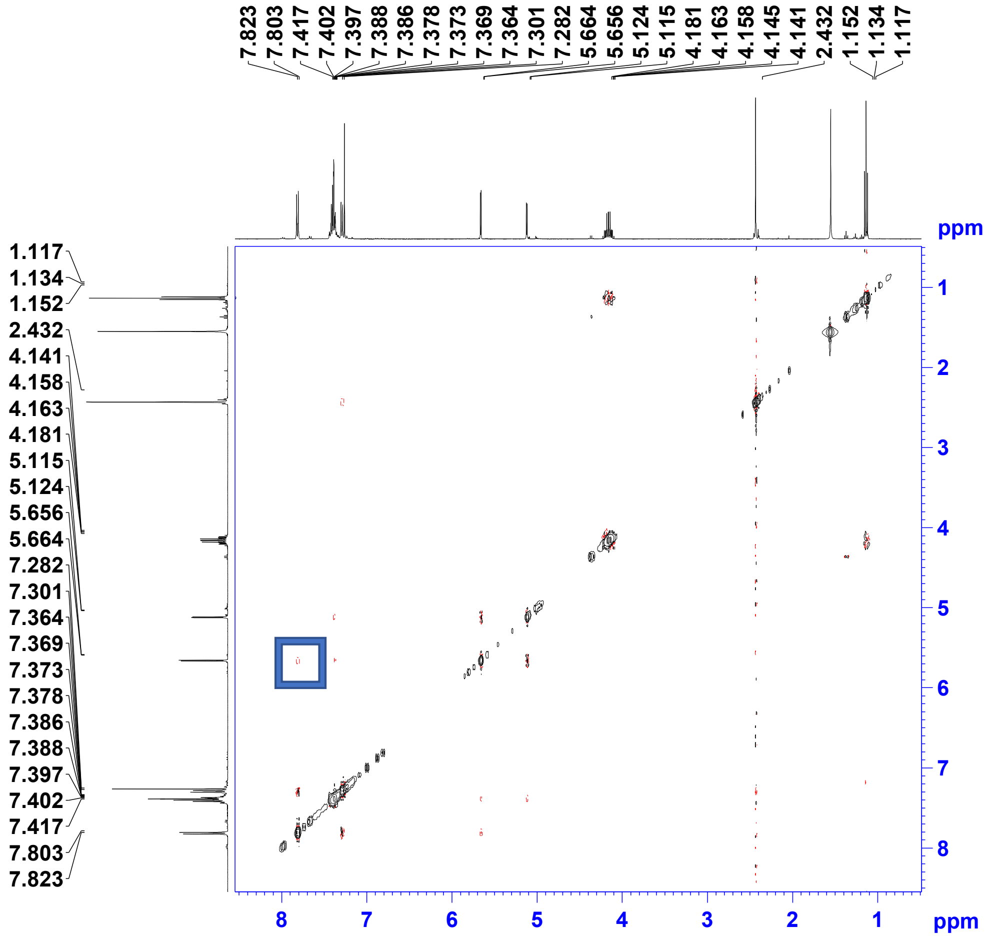
7.823
7.803
7.417
7.402
7.397
7.388
7.386
7.378
7.373
7.369
7.364
7.301
7.282
5.664
5.656
5.124
5.115
4.181
4.163
4.158
4.145
4.141
2.432
1.152
1.134
1.117
ppm
8
7
6
5
4
3
2
1
ppm
